# Timing of Debridement, Antibiotics, and Implant Retention for Early Periprosthetic Joint Infection: Data from the Finnish Arthroplasty Register

**DOI:** 10.2106/JBJS.25.00946

**Published:** 2025-11-19

**Authors:** Hannes Keemu, Eetu Syystö, Riku Klén, Mikko S. Venäläinen, Matias Hemmilä, Jutta Järvelin, Antti P. Eskelinen, Keijo T. Mäkelä

**Affiliations:** 1 Department of Orthopaedics and Traumatology, Turku University Hospital, and University of Turku, Turku, Finland; 2 Turku PET Centre, University of Turku, and Turku University Hospital, Turku, Finland; 3 Department of Medical Physics, Turku University Hospital, and University of Turku, Turku, Finland; 4 Finnish Institute for Health and Welfare, Helsinki, Finland; 5 Coxa Hospital for Joint Replacement and Faculty of Medicine and Health Technologies, University of Tampere, Tampere, Finland

## Abstract

**Background:**

Debridement, antibiotics, and implant retention (DAIR) is the method of choice in the treatment of acute periprosthetic joint infection (PJI). However, the optimal timing of DAIR is somewhat unclear. We assessed the success of DAIR performed during different time intervals after primary total hip arthroplasty (THA) and total knee arthroplasty (TKA) using data from the Finnish Arthroplasty Register (FAR).

**Methods:**

There were 178,498 primary operations (78,888 THAs and 99,610 TKAs) from May 2014 to April 2022 recorded in the FAR. Male patients represented 53.4% of the THA group and 55.5% of the TKA group. The most common age group was ≤62 years in the THA group and ≥76 years in the TKA group. All patients were of Finnish ethnicity. A total of 1,014 DAIR procedures were performed within 6 months after the primary arthroplasty. Cases of reoperation after DAIR were followed for 1 year after the DAIR; re-revision due to PJI within 1 year was regarded as a failure of the DAIR treatment. We compared the failure rate of DAIR among 3 time intervals: 0 to 42, 43 to 84, and 85 to 180 days after the primary operation. A Cox regression model was used to assess risk factors for re-revision.

**Results:**

In the THA group, the failure rate was 15.1% when DAIR was performed within 42 days, 10.0% when performed at 43 to 84 days, and 31.4% when performed at 85 to 180 days after the primary THA. In the TKA group, the failure rate was 8.9% when DAIR was performed within 42 days, 16.7% when performed at 43 to 84 days, and 9.8% when performed at 85 to 180 days after the primary TKA. Later DAIR was not associated with an increased re-revision risk, compared with the reference of 0 to 42 days, in the THA group (43 to 84 days: hazard ratio [HR], 1.2 [95% confidence interval (CI), 0.6 to 2.2; p = 0.63]; 85 to 180 days: HR, 1.4 [95% CI, 0.6 to 3.0; p = 0.41]). The same was true in the TKA group (43 to 84 days: HR, 1.0 [95% CI, 0.4 to 2.4; p = 0.98]; 85 to 180 days: HR, 1.9 [95% CI, 1.0 to 3.8; p = 0.065]).

**Conclusions:**

The failure rate of DAIR may not increase as much as previously thought if performed >6 weeks after primary total joint arthroplasty. Thus, DAIR can also be worth considering as a treatment method for PJI beyond the first 6 weeks postoperatively, depending on the severity of the case.

**Level of Evidence:**

Therapeutic Level III. See Instructions for Authors for a complete description of levels of evidence.

Early periprosthetic joint infection (PJI) is a serious complication that occurs at a rate of approximately 1%^1,2^. PJI may have disastrous consequences, and revision for PJI typically decreases the quality of life of the patient^3^. With the progressively increasing number of total hip arthroplasties (THAs) and total knee arthroplasties (TKAs) performed worldwide, the number of PJIs is expected to increase in the following years^2,4^.

PJIs can be classified into early infections (occurring within 3 months postoperatively), delayed infections (developing from >3 to 24 months postoperatively), and late infections (arising >24 months after surgery). According to most guidelines, the treatment of choice for acute postoperative PJI is debridement, antibiotic therapy, and implant retention (DAIR). The exchange of modular polyethylene components is recommended during DAIR^2^.

According to some previous studies, DAIR is effective when performed within 4 weeks after the primary arthroplasty^5^. However, it is still somewhat unclear how viable an option DAIR represents for treating PJIs with a more delayed presentation. The presence of a mature biofilm at later stages of the infection may decrease the effectiveness of DAIR. According to a recent systematic review, the success rate of DAIR for the treatment of PJI varied from 56% to 90% in single-clinic study settings, with various follow-up times, when DAIR was performed early^2^. In a 2-hospital study with a 1-year follow-up, the success rate was 84% when DAIR was performed during the first 3 months after THA and TKA and 47% after that^6^. In a study based on Dutch national registry data, there was no difference in re-revision rate during a 1-year follow-up between DAIR performed within 4 weeks and from 4 to 12 weeks^7^.

The Finnish Arthroplasty Register (FAR) was established in 1980, and became online-only in May 2014. Our primary aim was to use FAR data from 2014 to 2022 with 1-year follow-up after DAIR to compare the failure rate of DAIR among 3 time intervals: 0 to 42 days (0 to 6 weeks), 43 to 84 days (>6 to 12 weeks), and 85 to 180 days (>12 weeks to 6 months) after primary THA and TKA. Furthermore, risk factors for re-revision following DAIR were assessed.

## Materials and Methods

The FAR is a nationwide register that includes information on THA and TKA in Finland. Finnish health-care units are obligated to report arthroplasties to the FAR, which is maintained by the Finnish Institute for Health and Welfare^1^. Patient and surgery-related data such as body mass index (BMI) and surgical approach are reported to the FAR through a standard online sheet to which information is added during and immediately after the operation. Primary and revision arthroplasties are linked to each other through the use of a personal identification number. Dates of death are obtained from the Digital and Population Data Services Agency.

During the study period, the coverage of Finnish hospitals was 100%. The completeness of the primary THA and TKA data in the FAR, compared with the Care Register for Health Care, varied annually between 95% and 100%. The completeness of revision THA and TKA data varied annually between 82% and 92%^1^.

**Fig. 1:**
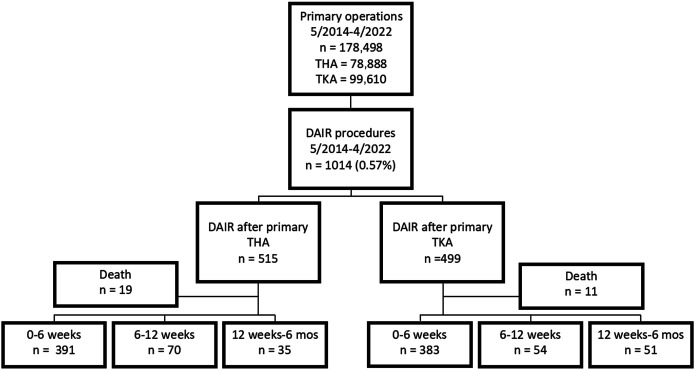
Flowchart of the study patients.

**Fig. 2:**
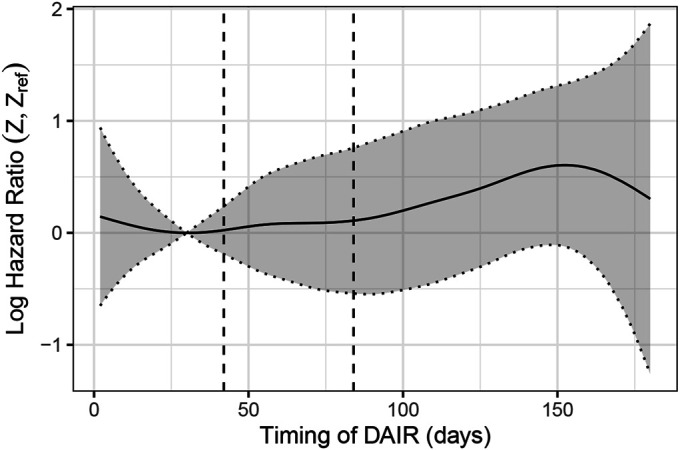
Nonparametric estimates of the dependence of the risk of re-revision on the timing of the DAIR. Estimates are presented as log HRs with 95% CIs with respect to a reference time point of 30 days (the minimum of the HR curve). The vertical dashed lines indicate the cutoffs used for grouping the patients (0 to 42, 43 to 84, and 85 to 180 days) for further analysis.

We included all primary THAs and TKAs performed in the period of May 2014 to April 2022. There were 178,498 primary operations (78,888 THAs and 99,610 TKAs) during that period with at least 1-year follow-up (Fig. 1). A total of 1,014 subsequent DAIR operations (after 515 THAs and 499 TKAs) for PJI or suspected PJI within 6 months were reported to the FAR during the same time period. There were 19 deaths in the THA group and 11 deaths in the TKA group registered during the follow-up after DAIR. DAIR was defined as deep (not superficial) irrigation and debridement of the joint with or without exchange of the femoral head and acetabular liner in THA and as exchange of the polyethylene insert in TKA. A re-revision performed for DAIR was our primary end point and was defined as any reoperation performed for PJI, such as repeat DAIRs and 1-stage and 2-stage revisions. We compared the failure rate of DAIR among 3 time intervals: 0 to 42, 43 to 84, and 85 to 180 days after the primary operation. Also, a separate time-to-event analysis using Cox proportional-hazards regression with spline smoothing was performed to enable a more detailed overview on the effects of DAIR timing on re-revision risk. By estimating a flexible hazard ratio (HR) curve for the timing of DAIR as a continuous variable, that analysis provided additional support for the selection of our discrete timing categories (Fig. 2).

A Cox regression model was used to assess risk factors for re-revision. The risk factors assessed were age group (≤62, 63 to 69, 70 to 75, ≥76 years), sex, American Society of Anesthesiologists (ASA) class (1 to 4), BMI (≤24, 25 to 29, 30 to 34, 35 to 39, ≥40 kg/m^2^), Charlson Comorbidity Index (1, 2, 3 to 4, ≥5), diagnosis (primary osteoarthritis, inflammatory arthritis, fracture, other), mode of anesthesia (spinal, general), and use of local infiltrative anesthesia (LIA) (yes, no). The Charlson Comorbidity Index was calculated on the basis of data in the Care Register for Health Care with which the FAR data were linked.

**Fig. 3:**
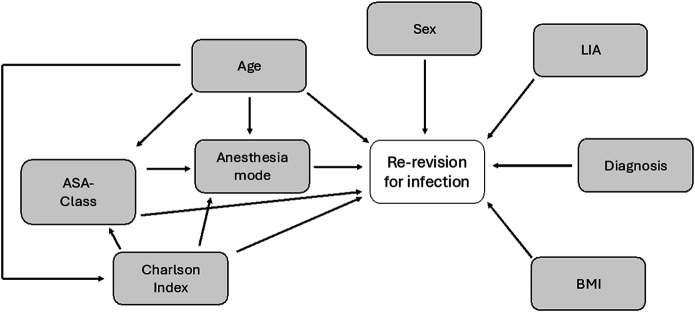
Directed acyclic graph (DAG) for the selection of covariates in adjusted Cox proportional-hazards regression analysis.

The statistical analysis was carried out using R (version 4.3.1; The R Foundation)^8^. Unadjusted and adjusted Cox proportional-hazards regression models were used to estimate HRs with 95% confidence intervals (CIs) for re-revision. Based on previous literature and clinical practice, we performed a directed acyclic graph (DAG) analysis (Fig. 3) to organize variables and their supposed relation to re-revision and other variables. The unadjusted Cox proportional-hazards regression model was then used to analyze the effects of potential risk factors for re-revision due to PJI by means of HRs and 95% CIs. All potential risk factors whose effects might be biased by confounders also underwent a Cox analysis in which they were adjusted on the basis of their relation to other factors on the DAG. The following 3 risk factors were adjusted on the basis of related covariates on the DAG: anesthesia (using age, ASA class, and Charlson index), ASA class (using age and Charlson Comorbidity Index), and Charlson Comorbidity Index (using age).

The fulfillment of the proportional-hazards assumption for the Cox models was assessed visually from the Kaplan-Meier curves and by using a test based on the scaled Schoenfeld residuals^8,9^. Some of the categories had only uncensored subjects (with no revisions), and, in those few cases, the models were not built. These categories were ASA class 1 and LIA in the THA group and BMI of ≤24 kg/m^2^ in the TKA group. For these variables, unadjusted models were not constructed for the particular joint. These variables were also omitted from the adjusted models for both joints. In addition, for the adjusted model for the TKA group, the variables of inflammatory arthritis and fracture were also dropped because the number of occurrences was too low. The flexible HR curve for the timing of DAIR as a continuous variable was generated using the R packages smoothHR and ggplot2. The level of significance was set at p < 0.05.

## Results

Demographic data of the patients who underwent DAIR are presented in Table 1. All patients were of Finnish ethnicity. Male patients represented 53.4% of the THA group and 55.5% of the TKA group. Patients with a BMI of ≥35 kg/m^2^ represented 21.9% of the THA group and 24.6% of the TKA group. The most common age group was ≤62 years in the THA group and ≥76 years in the TKA group.

**Table 1.** Characteristics at the Time of the Primary Procedure for the Patients Who Later Underwent DAIR*

Characteristic	THA Group (N = 515)	TKA Group (N = 499)
Age group		
≤62 years	139 (27.0%)	125 (25.1%)
63 to 69 years	133 (25.8%)	119 (23.8%)
70 to 75 years	119 (23.1%)	124 (24.8%)
≥76 years	124 (24.1%)	131 (26.3%)
Male sex	275 (53.4%)	277 (55.5%)
ASA class		
1	33 (6.4%)	24 (4.8%)
2	204 (39.6%)	209 (41.9%)
3 to 4	275 (53.4%)	263 (52.7%)
BMI		
≤24 kg/m^2^	60 (11.7%)	51 (10.2%)
25 to 29 kg/m^2^	157 (30.5%)	153 (30.7%)
30 to 34 kg/m^2^	154 (29.9%)	153 (30.7%)
35 to 39 kg/m^2^	89 (17.3%)	93 (18.6%)
≥40 kg/m^2^	24 (4.6%)	30 (6.0%)
Charlson Comorbidity Index		
1	99 (19.2%)	109 (21.8%)
2	73 (14.2%)	98 (19.6%)
3 to 4	104 (20.2%)	90 (18.0%)
≥5	85 (16.5%)	78 (15.6%)
Preoperative diagnosis		
Primary osteoarthritis	415 (80.6%)	458 (91.8%)
Inflammatory arthritis	7 (1.4%)	14 (2.8%)
Fracture	38 (7.4%)	27 (5.4%)
Other	55 (10.7%)	0 (0.0%)
Spinal anesthesia†	337 (65.4%)	353 (70.7%)
No LIA†	508 (98.6%)	485 (97.2%)

* The values are given as the number of patients, with the percentage in parentheses.

† Anesthesia mode and the use of local infiltrative analgesia (LIA) are presented for the DAIR; all others are presented for the primary procedure.

Of the 515 DAIRs performed after primary THA, 391 were performed during the first 42 days (6 weeks), 70 were performed at 43 to 84 days (>6 to 12 weeks), and 35 were performed at 85 to 180 days (>12 weeks to 6 months). Of the 499 DAIRs performed after primary TKA, 383 were performed during the first 42 days, 54 were performed at 43 to 84 days, and 51 were performed at 85 to 180 days (Fig. 1).

There were 77 re-revisions for PJI after THA and 48 re-revisions for PJI after TKA.

In the THA group, the failure rate was 15.1% (n = 59) when DAIR was performed within 42 days, 10.0% (n = 7) when performed at 43 to 84 days, and 31.4% (n = 11) when performed at 85 to 180 days after the primary THA. After TKA, the failure rate was 8.9% (n = 34) when DAIR was performed within 42 days, 16.7% (n = 9) when performed at 43 to 84 days, and 9.8% (n = 5) when performed at 85 to 180 days after the primary TKA.

In the Cox model, later timing of DAIR was not associated with an increased re-revision risk in the THA group, compared with the reference of 0 to 42 days (43 to 84 days: HR, 1.2 [95% CI, 0.6 to 2.2; p = 0.63]; 85 to 180 days: HR, 1.4 [95% CI, 0.6 to 3.0; p = 0.41]). The same was true in the TKA group (43 to 84 days: HR, 1.0 [95% CI, 0.4 to 2.4; p = 0.98]; 85 to 180 days: HR, 1.9 [95% CI, 1.0 to 3.8; p = 0.065]) (Table 2).

**Table 2.** Unadjusted Re-Revision Risk for Each Risk Factor in the Cox Model

Variable	THA Group		TKA Group	
	HR (95% CI)	P Value	HR (95% CI)	P Value
DAIR timing				
0 to 6 weeks	Reference		Reference	
>6 to 12 weeks	1.2 (0.6 to 2.2)	0.63	1.0 (0.4 to 2.4)	0.98
>12 weeks to 6 months	1.4 (0.6 to 3.0)	0.41	1.9 (1.0 to 3.8)	0.065
Age				
≤62 years	Reference		Reference	
63 to 69 years	1.1 (0.6 to 2.1)	0.72	1.1 (0.6 to 2.3)	0.73
70 to 75 years	0.9 (0.5 to 1.8)	0.84	1.0 (0.5 to 2.1)	0.99
≥76 years	1.2 (0.0 to 1.6)	0.65	0.7 (0.4 to 1.6)	0.46
Sex				
Male	Reference		Reference	
Female	1.1 (0.7 to 1.7)	0.63	0.5 (0.3 to 0.8)	0.01
ASA class				
1	Reference		Reference	
2	1.8 (0.4 to 7.5)	0.44	0.7 (0.2 to 2.5)	0.64
3 to 4	3.4 (0.8 to 13.8)	0.09	1.0 (0.3 to 3.3)	0.97
BMI				
≤24 kg/m^2^	Reference		Reference	
25 to 29 kg/m^2^	1.7 (0.6 to 4.5)	0.29	2.4 (0.5 to 10.5)	0.25
30 to 34 kg/m^2^	2.3 (0.9 to 5.9)	0.09	4.4 (1.0 to 18.7)	0.04
35 to 39 kg/m^2^	1.8 (0.6 to 5.1)	0.26	3.5 (0.8 to 15.7)	0.1
≥40 kg/m^2^	4.0 (1.3 to 12.7)	0.017	1.8 (0.2 to 12.6)	0.57
Charlson Comorbidity Index				
1	Reference		Reference	
2	1.8 (0.8 to 4.1)	0.16	0.7 (0.3 to 1.6)	0.40
3 to 4	2.4 (1.1 to 5.0)	0.02	1.5 (0.7 to 3.1)	0.32
≥5	1.6 (0.7 to 3.6)	0.29	0.9 (0.4 to 2.1)	0.77
Anesthesia				
Spinal	Reference		Reference	
General	1.6 (1.0 to 2.5)	0.05	1.2 (0.7 to 2.1)	0.48
LIA				
No	Reference		Reference	
Yes	1.0 (0.1 to 7.1)	0.99	2.8 (1.0 to 7.6)	0.05
Diagnosis				
Primary osteoarthritis	Reference		Reference	
Inflammatory arthritis	0.7 (0.2 to 3.0)	0.66	<0.001 (0 to infinity)	0.99
Fracture	1.3 (0.6 to 2.8)	0.52	0.9 (0.3 to 2.9)	0.86
Other	1.4 (0.8 to 2.4)	0.24	0.9 (0.3 to 2.9)	0.86

In the additional time-to-event analysis treating the timing of DAIR as a continuous variable, re-revision risk again did not change significantly over time (Fig. 2).

Female sex was associated with a decreased risk of re-revision after DAIR in the TKA group (HR, 0.5 [95% CI, 0.3 to 0.8; p = 0.007]). Superobese patients with a BMI of ≥40 kg/m^2^ had an increased risk of re-revision after DAIR in the THA group (HR, 4.0 [95% CI, 1.3 to 12.7; p = 0.017]) (Table 2). In the adjusted Cox model, general anesthesia was associated with an increased risk of re-revision after DAIR in the THA group (HR, 2.0 [95% CI, 1.2 to 3.5; p = 0.008]). A Charlson Comorbidity Index of 3 to 4 was associated with an increased risk of re-revision after DAIR in the THA group (HR, 2.4 [95% CI, 1.2 to 5.1; p = 0.02]) (Table 3).

**Table 3.** Adjusted Risk Factors for the Risk of Re-Revision in the Cox Model Based on the DAG*

Variable	THA Group		TKA Group	
	HR (95% CI)	P Value	HR (95% CI)	P Value
ASA class				
1	Reference		Reference	
2	0.8 (0.1 to 6.4)	0.83	0.4 (0.05 to 3.0)	0.36
3 to 4	2.4 (0.3 to 18.4)	0.40	0.5 (0.07 to 4.2)	0.55
Anesthesia				
Spinal	Reference		Reference	
General	2.2 (1.3 to 3.7)	0.003	1.3 (0.7 to 2.5)	0.42
Charlson Comorbidity Index				
1	Reference		Reference	
2	1.8 (0.8 to 4.1)	0.16	0.7 (0.3 to 1.7)	0.41
3 to 4	2.4 (1.2 to 5.1)	0.02	1.4 (0.7 to 3.0)	0.36
≥5	1.6 (0.7 to 3.7)	0.25	0.9 (0.4 to 2.1)	0.77

* ASA class was adjusted for age group and Charlson Comorbidity Index. Anesthesia mode was adjusted for age group, ASA class, and Charlson Comorbidity Index. Charlson Comorbidity Index was adjusted for age.

## Discussion

We found that the failure rate of DAIR may not increase as much as previously thought if performed >6 weeks after primary total joint arthroplasty. In the Cox model, later timing of DAIR was not associated with an increased re-revision risk, both in the primary analysis and when time was considered as a continuous variable in a secondary analysis. Female sex was associated with a decreased risk of re-revision after DAIR in the TKA group. Superobese patients with a BMI of ≥40 kg/m^2^ had an increased risk of re-revision after DAIR in the THA group. According to our data, DAIR can also be worth considering as a treatment method for PJI beyond an early 6-week time period, depending on the severity of the case.

According to previous studies, DAIR is very effective when performed early after a primary arthroplasty. In a single-center study with a 42-month mean follow-up of 38 patients treated with DAIR, Barros et al. reported that the overall success rate was 89% when DAIR was performed at a mean of 23 days (range, 6 to 30 days) after primary THA or TKA^5^. Tirumala et al. assessed 103 culture-positive PJIs that were treated with DAIR within 4 weeks after the primary procedure and found a success rate of 88% at 1 year^10^.

However, it had still been somewhat unclear how viable an option DAIR represents for PJIs with a more delayed presentation. In a 2-hospital study from The Netherlands with 84 patients and 1-year follow-up, the success rate of DAIR after THA and TKA was 84% when performed during the first 3 months but only 47% when performed after that^6^. In a single-center study with a mean 4-year follow-up, Sendi et al. assessed 46 hips that underwent DAIR; of the hips that had received a PJI diagnosis within 3 months after THA, the successful outcome rate was excellent at 90%^11^. However, in a single tertiary clinic setting, Chalmers et al. reported that the success rate of DAIR for a PJI diagnosis within 3 months after THA or TKA was only 58% by 2 years; in that study, DAIR was always performed within 3 weeks after symptom onset^12^. In 6 hospitals in 4 countries (United States, Spain, Portugal, and The Netherlands), Löwik et al. assessed 769 patients with 1-year follow-up after DAIR performed within 90 days of the index arthroplasty^13^. The treatment failure rate was 42% for DAIR performed in weeks 1 to 2, 38% for weeks 3 to 4, 29% for weeks 5 to 6, and 42% for weeks 7 to 12. However, the exchange of modular components occurred in only approximately one-half (40% to 64%) of the cases, and a polymicrobial finding was common (35% to 42%). Furthermore, methicillin-resistant *Staphylococcus aureus* (MRSA), *Staphylococcus epidermidis,* Enterococcus, and Pseudomonas were often involved. The authors concluded that DAIR can be performed late, but that it is important to do it within 1 week after the onset of symptoms.

It is obvious that the diagnostic criteria for PJI, antibiotic regimen, length of follow-up, time from symptom onset to DAIR, technical performance of the DAIR, and microorganisms present varied considerably among the studies and certainly can account for many of the differences. The proportion of MRSA and other bacteria that are difficult to treat using DAIR is important information. Single-center studies or studies performed in only a few centers are usually able to provide these granular data. Also, the completeness of information on the performance of revision operations is usually high in single-center studies, but the number of PJIs can be small.

Our study had several limitations. One major limitation of our national arthroplasty registry study was that our data did not contain microbiology or antibiotic regimen data or the time from symptom onset. A delay exceeding 1 to 2 weeks from the onset of symptoms may lead to substantially lower rates of DAIR success. In Finland, there have been only a very limited number of PJIs caused by methicillin-resistant bacteria so far, which may partially explain the relatively high success rate of DAIR in the current study.

A second limitation of our study was that a diagnosis of PJI in the FAR is based on the clinical decision-making of the orthopaedic surgeon in the emergency department, in policlinics, and in the operating room (clinical and laboratory findings). Electronic input of the data into the FAR is performed in the operating room. Unfortunately, the bacterial culture result is often not ready at this stage. Thus, the FAR’s PJI definition is based on clinical findings, and it is not corrected later when the bacterial culture result becomes available. Furthermore, a PJI diagnosis in the FAR is not further classified as acute, subacute, or chronic, but only as a PJI. Therefore, we did not have information on the exact clinical presentation of the PJI cases (pus in aspirate or not).

A third limitation of our study was that the completeness of revision THA or TKA in the FAR was somewhat lower than that of primary surgery. Revisions performed on call, such as revisions for PJI, may be especially underreported. During some of the study years, any information about the revisions was absent in 18% of procedures. This should be taken into account when interpreting our results. However, we believe that the distribution of missing revisions is probably independent of the time since the primary surgery, so there should not be a major bias in comparisons of success rates among different time periods.

A fourth limitation of our study was that, in rare cases, long-term antibiotic suppression or even lifelong antimicrobial therapy may have been used instead of revision surgery. Permanent antibiotic treatment can be interpreted as failed infection treatment, but, unfortunately, we were unable to detect those unrevised cases. Furthermore, we were unable to determine if some of the late PJIs were actually acute hematogenous infections, although the treatment regimen would be the same. Also, the follow-up time (1 year) in our study was relatively short. There will certainly be some PJI relapses after that time period. However, 1-year follow-up has also been used previously^7,14^.

A strength of our study was the large number of DAIR procedures and re-revisions compared with previous studies.

Van der Ende et al. assessed 514 DAIR procedures after THA or TKA using Dutch national registry data. In the 1-year follow-up, there was no difference in re-revision rates between DAIR performed <4 weeks postoperatively (20% for THA and 21% for TKA) and from 4 to 12 weeks postoperatively (17% for THA and 20% for TKA)^7^. Our results give support to the Dutch results. However, the time periods of the 2 studies were not directly comparable, because we also had a special interest in DAIR performed >3 to 6 months after primary THA or TKA. Furthermore, the completeness of revision surgery capture in the Dutch registry may be higher than that in the FAR^15^.

In Finland, modular components are always exchanged if possible, which certainly helped in obtaining a successful outcome. Arthroscopic lavage is not used in our country.

The success rate of DAIR performed at >3 to 6 months was also acceptable in our study. We believe that DAIR may be considered instead of 1-stage or 2-stage revisions to treat late PJI in some patients, after careful consideration of patient and bacteriological status. DAIR has also been reported to have a 68% success rate at 2 years even when performed after revision arthroplasty^1^.

We were able to assess the Charlson Comorbidity Index on the basis of the Care Register for Health Care. A Charlson Comorbidity Index of 3 to 4 was associated with an increased re-revision risk after DAIR performed after primary THA but not after primary TKA. However, the number of re-revisions in our study was relatively small, which may have prevented finding a significant result for TKA. A high BMI of ≥40 kg/m^2^ was associated with an increased re-revision risk after DAIR performed after primary THA in our study, although Fink et al.^16^ and de Vries et al.^6^ did not find any such association.

According to van der Ende et al.^7^, male sex was a risk factor for re-revision after DAIR performed after THA (odds ratio, 3.1). Likewise, male sex was a risk factor for re-revision in our study, especially after DAIR performed after primary TKA. Male sex is a known risk factor for developing PJI after THA or TKA^17,18^, and it now appears that the risk of treatment failure is also considerably higher in male patients, although not all studies^6^ have confirmed that. Higher ASA class, which is a crude estimate of patient comorbidity, was not a risk factor for re-revision in our study or in the Dutch study^7^.

In conclusion, the failure rate of DAIR may not increase as much as previously thought if performed >6 weeks after primary total joint arthroplasty. DAIR can thus also be worth considering as a treatment method for PJI beyond the first 6 weeks postoperatively, depending on the severity of the case.
